# Gaining a better understanding of respiratory health inequalities among cities: An ecological case study on elderly males in the larger French cities

**DOI:** 10.1186/1476-072X-12-19

**Published:** 2013-04-10

**Authors:** Christina Aschan-Leygonie, Sophie Baudet-Michel, Hélène Mathian, Lena Sanders

**Affiliations:** 1 UMR Environnement Ville Société, Université de Lyon, Faculté GHHAT, 5 avenue Pierre Mendès-France, Bron Cedex 69676, France; 2 UMR Géographie-cités, CNRS, Universités Paris I Sorbonne, Paris VII Diderot, 13 rue du Four, Paris 75006, France

**Keywords:** Respiratory health, Chronic obstructive pulmonary disease, Cities, Socioeconomic characteristics, Air pollution, Physical characteristics, Regional context, Intra-urban organization, Scale, France

## Abstract

**Background:**

In recent years, there have been a growing number of studies on spatial inequalities in health covering a variety of scales, from small areas to metropolitan areas or regions, and for various health outcomes. However, few investigations have compared health status between cities with a view to gaining a better understanding of the relationships between such inequalities and the social, economic and physical characteristics. This paper focuses on disparities in respiratory health among the 55 largest French cities. The aim is to explore the relationships between inter-urban health patterns, city characteristics and regional context, and to determine how far a city’s health status relates to the features observed on different geographical scales.

**Methods:**

We used health data describing hospitalizations for Chronic Obstructive Pulmonary Disease (COPD) as a proxy for respiratory health, and the total number of hospitalizations (overall) as a proxy for general health. This last indicator was used as a benchmark. A large set of indicators relating to socioeconomic, physical and amenity aspects of the cities (urban units) was also constructed. Data were analyzed using linear correlations and multiple linear regression models.

**Results:**

The results suggest that socioeconomic characteristics are major discriminators for inequalities in respiratory health status among urban units. Indeed, once combined to socioeconomic characteristics, only a climate indicator remained significant among the physical indicators. It appeared that the pollution indicators which were significantly correlated with COPD hospitalization rates loosed significance when associated to the socio-economic indicators in a multiple regression. The analysis showed that among the socio-economic indicators, an employment indicator derived at the regional scale, and two indicators reflecting the unequal intra-urban spatial distribution of population according to their education, were the most efficient to describe differences in the respiratory health status of urban units.

**Conclusion:**

In order to design effective urban policies, it is essential to gain a better understanding of the differences among cities in their entirety, rather than solely differences across small urban areas or individuals.

## Abstract (fr)

Un nombre croissant d’études sur les inégalités spatiales de santé, conduites à des échelons très différents, des quartiers aux aires métropolitaines ou aux régions a été publié au cours des années récentes. Ces études couvrent des pathologies variées. Peu d’études ont cependant adopté une approche comparative et ont eu pour ambition d’expliquer les différences interurbaines de santé en mobilisant à la fois des indicateurs sociaux, économiques et physiques. Cet article propose une étude de cas sur les disparités interurbaines de santé respiratoires parmi les 55 plus grandes villes françaises. L’objectif ici est d’explorer les relations entre les différenciations interurbaines de santé et les caractéristiques des villes, en tenant compte de ces caractéristiques à différents échelons (ville, région, quartier).

**Méthodes**

Nous avons mobilisé une information sur les hospitalisations pour Broncho Pneumopathie Chronique Obstructive (BPCO) pour approcher la situation respiratoire des villes et sur l’ensemble des hospitalisations pour approcher la situation de santé générale de ces mêmes villes. Un grand nombre d’indicateurs socio-économiques, d’indicateurs décrivant l’environnement physique et les équipements de soins ont été construits pour décrire les villes. L’information a été analysée en utilisant des régressions linéaires simples et multiples.

**Résultats**

Les résultats suggèrent que les caractéristiques socio-économiques sont des facteurs majeurs de différenciation entre les villes du point de vue des situations de santé. Combinées aux indicateurs socio-économiques, seul l’indicateur des températures de janvier reste significatif. Les indicateurs de pollution, qui sont significativement associés aux taux d’hospitalisation pour BPCO, perdent de leur pouvoir explicatif lorsqu’ils sont associés aux indicateurs socio-économiques dans la régression multiple. Les résultats montrent que parmi les indicateurs économiques, le niveau de chômage régional s’associe à des indicateurs reflétant l’inégale répartition des populations, selon leur niveau d’éducation, à l’intérieur de la ville pour décrire de manière efficace les différences interurbaines de santé respiratoire.

Afin de mieux réfléchir aux politiques de santé, il paraît important de s’intéresser aussi aux aspects collectifs des différences interurbaines de santé et pas uniquement aux différences entre individus.

## Introduction

Epidemiological studies seek to identify and isolate factors that explain health inequalities in time and space, or among individuals. This study focuses on the spatial dimension of health inequalities. There are different epistemological ways of studying the relationship between health and space. One approach is to observe the individual level in order to assess how far the social, economic and ecological characteristics of a place influence the health of its residents. Researchers adopting this kind of approach often use multilevel models. A second approach focuses on the health inequalities among different places and the resulting spatial patterns. The aim is then to identify the relationships between health patterns and the spatial structure of different social, economic and environmental features.

The research work presented in this paper is based on the second approach. It is thus an ecological study, and examines differences in respiratory health across cities. The aim is to explore the relationships between the respiratory health situation of different cities and their medical amenities, socio-economical and physical characteristics. Intra-urban differences are also considered among the characteristics that can contribute to the overall respiratory health status of a city. Various geographical scales are used in order to derive information on these characteristics: the regional scale, defining the context in which a city is located and that will be referred to as the *regional context;* the city scale; and finally the intra-urban scale, used to characterize the intra-urban organization of a city.

Our investigation does not focus on the individuals ^a^ themselves. In this study cities are the objects of our study. They are not simply considered as aggregates of individuals but regarded as functionally coherent spatial entities, in the spirit of “cities as systems within system of cities” [1]. Indeed, as pointed out by Cummins et al. [2] we need to understand the complex and interdependent factors associated with spatial health variations in order to design effective “contextually sensitive” policy interventions to improve public health in cities. Our choice of focusing on the city level is one way of achieving this goal.

The paper focuses on Chronic Obstructive Pulmonary Disease (COPD) which is a major cause of chronic morbidity (44 millions of patients diagnosed worldwide in 2006) and mortality worldwide [3]. According to WHO estimates for 2030 [3], COPD is predicted to become the third leading cause of death in the world. Among respiratory diseases, COPD is the most highly correlated with air pollution and its relation to urban pollutants is well documented in studies throughout Europe [4-7]. Other studies in the US [8-10] or New-Zeeland [11] have shown that mortality among COPD patients is directly related to a particulate rise in the atmosphere. Here we present results concerning elderly males since this age group is considered particularly vulnerable to COPD pathology [7,12,13]. The link between urban air pollution and COPD is well established in time: authors look at daily relations between urban particulate concentration and COPD mortality or morbidity. However there are no analysis developing a comparative approach of differences in COPD morbidity among places, such as cities.

## Background

### The spatial approach in health research

There is some methodological debate about how to approach the relationship between place and health [14-16]. Some authors have pointed out the lack of a firm theoretical framework for understanding the processes underpinning this relationship [17,18].

In epidemiological studies, space can be regarded in two different manners. First, it can be considered as one of many potential factors explaining health differences. In this case, space refers to the socioeconomic and physical characteristics of the area in which the individuals studied are located. Different approaches can coexist and those that are the most commonly used are based on a multilevel modeling. In the second case, the spatial health inequalities themselves constitute the main subject of the research. The aim is then to understand how other geographical patterns are related to spatial health differentiations.

In most multilevel studies, the researchers investigate the effects of a place on an individual’s health, making a distinction between *compositional* and *contextual effects*. According to Macintyre et al. [17], there are compositional effects when health differences between places are attributable to the under- or over-concentration of individuals (classified according to age, social status, type of employment, etc.) with a specific health profile. There is less consensus on the terminology pertaining to contextual effects. The term *context* is generally related to amenities and opportunity structures in an individual’s environment. It concerns both the local physical characteristics in the area of residence (air or water quality, features of public spaces such as the presence of parks and road networks) and the extent of availability of public or private services in these areas, or again the sociocultural and historical features of the local communities, such as social cohesion [2,17].

In ecological studies, the focus is on spatial aggregates. As Curtis and Jones [16] have pointed out, the individual is not always the most relevant unit of analysis, and the small-area or regional level are more pertinent for some issues. Three types of ecological studies can be distinguished according to the level of observation:

#### Classic ecological studies, which investigate spatial differences in people’s health at a single level of observation

Often, studies of this type are based on the hypothesis that deprivation differences between areas account for a large part of spatial health variations. For example, Ben-Shlomo et al. [19] demonstrated that male and female mortality rates before age 65 in England were strongly associated with deprivation (Townsend index) and deprivation inequalities on ward and local authority level. Other explanatory factors concern the physical characteristics of the observed area. Corburn et al. [20], for instance, focusing on small areas of New-York, examined the effects of the built-up environment, housing quality, air pollution sources and noxious land use on intra-urban childhood asthma hospitalization rates.

#### Studies that take into account a geographical level higher than that of the object of study

Indeed, issues relating to compositional and contextual effects remain relevant when one considers the meso-level of spatial entities: a larger geographical entity, within which the observed spatial entity is located, can be regarded as its context. For example, Congdon et al. [21] studied health and mortality differences between wards in England and Wales. They considered the districts as spatial contexts for the wards and showed there were significant contextual effects for explaining the differences observed between wards with similar socioeconomic profiles. In the same manner, Phillimore and Morris [22] showed that small areas with similar deprivation levels had different mortality rates depending on their regional location, when the regions concerned exhibited different overall levels of deprivation. Their hypothesis was that differences in the provision and use of health services, in the levels of atmospheric pollutants and in the types of built-up environment could explain these health differences. Although they did not explicitly use the term “context”, they did refer to differentiation referring to a higher geographical level, which in many studies is considered as a contextual aspect.

#### Studies that take into account a geographical level lower than that of the object of study

A number of authors have used an ecological approach to consider how far residential segregation can explain differentiation in health outcomes. Most of such research focuses on the relationship between ethnic residential segregation and mortality in general, but there are also studies on the relationship between socioeconomic residential segregation and the incidence of specific illnesses such as tuberculosis and heart disease [23-25]. For instance, in a study involving 47 US cities, Cooper [26] examined cardiovascular mortality among black, Asian and white men and women. The author used aggregated data at city level. Residential segregation in the cities was characterized using a Gini coefficient. Cooper [26] showed that, independently from mean income variations across cities, intra-urban racial residential segregation was associated with higher rates of death from cardiovascular disease. Some authors, for example Acevedo-Garcia or Subramanian [27,28], also interpret residential segregation as a context that affects the health of individuals.

### Aims and hypotheses

The aim of the present study was to investigate inter-urban differences in respiratory health status across the 55 larger French cities (more than 100,000 inhabitants), with particular emphasis on two related issues:

What combination (if any) of socioeconomic and physical factors is associated with these differences?

Is a city’s health status related to both its intra-urban spatial organization (with possible segregation) and its regional context?

**Figure 1:**
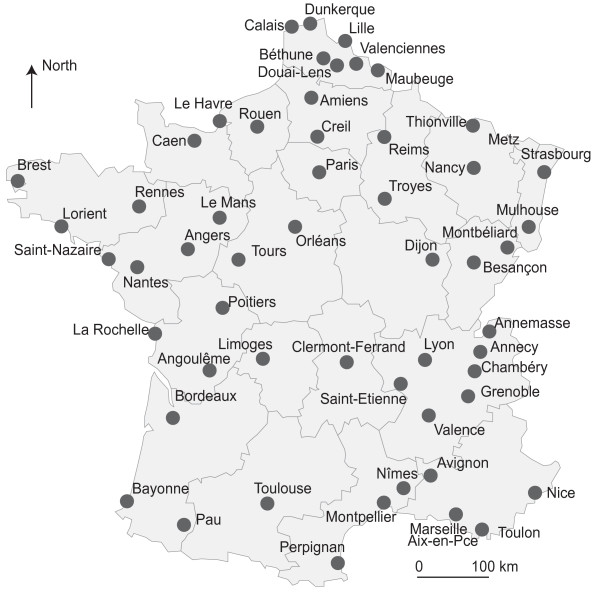
Largest French urban units: more than 100,000 inhabitants (2008).

Our first assumption is that a city is a geographical object whose physical and socioeconomic characteristics interact with each other to give rise to a specific respiratory health status of the city as an entity. The second assumption is that a city’s intra-urban organization, i.e. spatial organization patterns, likewise contributes to forming the respiratory health status of the city as a whole. The third assumption is that a city’s location in a regional context also contributes to its respiratory health status. Therefore the physical and socioeconomic characteristics need to be considered on different geographical scales (Figure 1):

**Figure 2:**
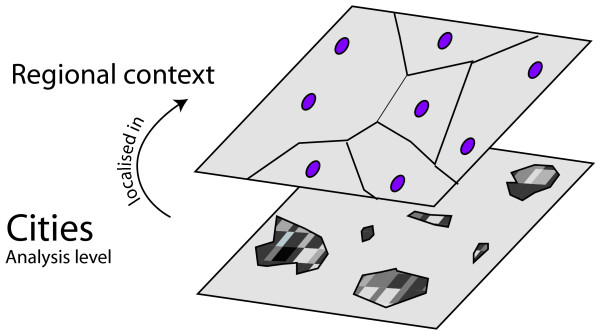
Scales of observation: the regional and city scale.

that of the city with, on the one hand, its socioeconomic features (social groups, educational levels, health amenities) and its physical features (local climate, air pollution, etc.), and on the other hand, its intra-urban organizational features, both socioeconomic and physical. The hypothesis to be examined is for example that two cities with similar socioeconomic and physical characteristics but different degrees and forms of intra-urban residential organization (Figure 2) might differ in terms of respiratory health status.

that of the region, where physical and socioeconomic profiles can be considered as proxies for an overall regional context. The hypothesis to be explored is that two cities with the same socioeconomic and physical characteristics but situated in different regional contexts may not have the same respiratory health profiles.

**Figure 3:**
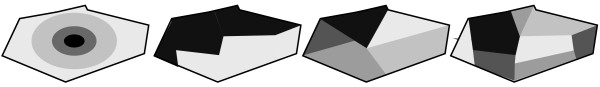
The intra-urban scale: patterns of spatial organization.

The objective of this paper is to analyze the relationship between the urban health status, measured for a particular pathology and for each city, and its physical and socioeconomic characteristics, taking into account the regional context and the intra-urban organization (Figure 3)^b^.

## Methods

In order to work with comparable urban entities, we chose to use urban units to define cities*.* Indeed three delimitations can be used in France in comparative urban studies [29]: the urban central municipality, the urban unit^c^ and the functional urban area^d^. The urban central municipality, corresponding to a political grid, is not suited to epidemiological questions. Urban units and functional urban areas both offer relevant delimitations for socioeconomic comparison. The functional urban area, encompassing rural, suburban and urban types of space, covers very different types of physical environment and is therefore not suitable. The urban unit, based on morphological continuity (less than 200 meters separating buildings), covers exclusively urban built-up areas and is well suited to a comparative perspective. As air pollution monitoring is obligatory for urban units with more than 100,000 inhabitants, the 55 French urban units exceeding this threshold were included in the study.

In order to apprehend the intra-urban organization, we used the IRIS census subdivisions. These census tracts are statistical units defined by INSEE, and known as IRIS (Ilots Regroupés pour des Indicateurs Statistiques). They comprise between 1,800 and 5,000 inhabitants. It is common to use this subdivision in France in segregation and socio-spatial urban studies [30,31].

### Data collection

Literature on the geography of respiratory health has been looking at respiratory mortality or hospitalization for causes such as lung cancer, COPD or asthma [32-39]. Authors investigating the link between physical characteristics, and more specifically pollution and health within cities either look at COPD or cardiovascular diseases [40,41]. Some authors concentrate on respiratory health within cities and most often look at asthma and/or COPD [4,6,42-46]. We used the incidence of hospitalization of elderly males as a proxy for the respiratory health in a city. This data used to create proxies for the urban unit health status were derived from the PMSI^e^ database which gathers information on all hospitalizations, comprising the patient’s place of residence (ZIP code), age, gender, admission and discharge dates and all diagnoses. COPD is a chronic limitation or obstruction of airflow [12,47] and it has been documented as a respiratory disease that is exacerbated by atmospheric pollution [5-7,10,48]. It is often considered as an adult (over 25) disability [34] and is more frequently diagnosed in males than females. Prevalence studies usually consider adults over 20 [12,33,47]. Jeannin [13] states that the first COPD hospitalization generally occurs between ages 65 and 69. Halonen et al. 2013 [7] linked COPD hospital emergency room visits by age group (children, adults and elderly > 64 years) with air pollution levels and showed a clear effect between COPD hospitalizations of elderly and particulate air pollution. Their findings also suggest that the mechanisms of respiratory effects of air pollution differ by age group. Therefore a study concentrating on only one age group can be interesting. According to the results of Tissot-Dupont [49] elderly people (> 60 years) are particularly vulnerable to respiratory infectious pathologies. For the present study 12 diagnostic codes describing COPD were chosen according to the Furham and Delmas [33] methodology. Data pertaining to COPD diagnoses for the year 2008 were extracted to identify and assess specific respiratory health patterns. In an initial stage we concentrated on all males over 65 (38,323). Several health indicators were tested: hospitalization rates by age group (65–70; 71–75; 75–80; 80–85; 86–90) and standardized hospitalization ratio (based on standardized mortality ratio methodology) for all the age groups pre-cited population. The 65–75 age group is considered to be one of the most vulnerable [12] for COPD. In the PMSI database, the 71–75 age group is more numerous than the 65–70 (8,340 versus 6,463), and the hospitalization ratio is higher (19 versus 15 per 1,000). Inter-urban patterns for elderly male COPD hospitalization rates are very similar to those provided by other COPD health indicators (age groups rates and standardized hospitalization ratio). Therefore elderly (age 71–75) males’ COPD hospitalization rates was chosen as indicator to characterize the respiratory health status of an urban unit. The same indicators were calculated for all hospitalizations whatever the diagnosis, and this overall hospitalization rate among elderly males served as a benchmark that enabled us to highlight the specific nature of respiratory health patterns (Additional file 1: Indicators and measurements).

A review of the literature on health and socioeconomic inequalities [35,42,43,46,50-55] pointed in the direction of a large number of indicators relating to various socioeconomic, physical and amenity aspects. For the socioeconomic dimension, classic indicators such as unemployment rates by age group [17,56-59], the proportion of individuals with no qualifications or those having attended at least two years of higher education [56,59-62], non-taxable household income were established for different scales of observation (Additional file 1: Indicators and measurements). INSEE^f^ socioeconomic databases dating from 2006 were used to create this set of indicators. Family physicians and pulmonologists indicators^g^ were also established to take into account the presence of one important type of health care amenity [63,64].

In order to characterize the intra-urban organization of the urban units in terms of residential segregation, three complementary indicators [65,66], were established: the coefficient of variation, which was used to represent the degree of heterogeneity across census tracts^h^ in the urban unit; the Gini concentration ratio, assessing the evenness of the spatial distribution of a given characteristic, such as educational level, across census tracts; and Moran’s spatial autocorrelation coefficient, which significance level values were used to measure the degree of similarity across census tracts. Based on literature review [67-69], educational characteristics are more appropriate for the French case than ethnic variables.

A large number of studies have confirmed that respiratory diseases are related to the physical characteristics of the living area: COPD has multi-factorial etiology, including exogenous factors like air pollution, cold [70] and topography [71-73]. Little is however known about the long term effects of climate, but there is evidence of statistical associations between the prevalence of respiratory diseases in different locations and various climatic factors or climate zones [11,74-77].

Recent scientific papers have discussed the link between meteorological and climatic factors and COPD prevalence [78]*.* Across France, because of its geographical situation and its rather large surface area (674,800 square kilometers), there is significant spatial variability in climate. Various climatic zones occur across the country (oceanic, semi-continental, mountain, mediterranean). It is therefore reasonable to hypothesize that climate can affect inter-urban spatial patterns of COPD prevalence. The effect could be direct, for example, an effect of air temperature or humidity on the reactivity of the respiratory tract. The effect could also be indirect through a differential exposure to air pollutants, infections or aeroallergens. If this is the case, spatial patterns of hospital admissions for COPD might be related to climatic zones and variability in climatic parameters like temperature or humidity. The climatic parameters considered in the literature vary: annual mean or seasonal mean temperature [11,74-77,79], mean temperature for the coldest and hottest months [75], annual variation of mean temperature [76,77,79], relative outdoor humidity [74,76,80,81] frequency of fog [11], and wind force or direction [11,80]. This review of the literature on climatic factors and their potential effects on respiratory health (most papers focus on asthma) led us to choose a wide array of indicators describing the urban units. Firstly, each urban unit was assigned to a climate zone which constitutes its regional climatic context. On urban unit scale, the following parameters were chosen: minimum and maximum temperatures in January and July, annual mean outdoor relative humidity minima, average number of foggy days per year (visibility less than one kilometer), average number of days with strong winds per year (wind > 57 km/h) and average number of hot days per year (> 25°C) were selected. The last parameter was chosen because there is clear evidence in the literature reporting temporal studies that high temperature events (heat waves) increase morbidity in general, and in particular for elderly people [82,83]. All these variables were derived from Météo-France meteorological data for the period of 1981–2010.

Two indicators defining the altitude of each urban unit were also included: firstly the altitude of the urban unit center. There is evidence that COPD prevalence is linked to altitude [71-73,84], but there are diverging results. Some studies suggest that higher altitude is associated with higher COPD prevalence [71,72,84], whereas others suggest the reverse relationship [73]. Secondly, in addition to the altitude of the urban unit centre, a measure of intra-urban variation in altitude was also introduced. There is no direct effect of altitude variability on COPD prevalence related in the scientific literature*.* This indicator was included because it might have an indirect link with COPD prevalence via effects on climatic parameters.

Some studies have investigated associations between daily variations in airborne pollen concentrations and respiratory morbidity or mortality [6,85-87]. These results indicate a positive relationship between daily rates of COPD mortality and airborne pollen concentrations. Their findings suggest that other small particles of biological origin can potentially have inflammatory effects and exacerbate COPD symptoms. In order to take account of the aerobiological characteristics of the urban units, RNSA^i^ pollen risk indexes were included. For each pollen type, RNSA defines a level of allergic risk and its geographical extension. The mapping of the situation in 2008 was used to construct a global index, derived from aeroallergen species^j^ risk levels. This index was established for each urban unit. Only pollens classified as medium, high or very high allergen risk by RNSA in 2008 were considered.

According to the literature there is strong evidence that pollutants exacerbate COPD symptoms [4,6,7,39,88-92]*.* Indicators of air pollution were created on the scale of the urban unit. Two ambient air pollutants, NO_2_, and PM_10_ were used. The dataset used for assessing the air pollutant levels for each pollutant in each urban unit is derived from ADEME^k^ and the Geovariances interpolation model. The ADEME-Geovariances model was developed from concentrations measured in urban background stations and estimated emission data [93]. The model estimates a range of pollutant levels: NO_2_ (annual mean of daily concentrations; annual mean of daily 95th percentile concentrations; mean of daily winter concentrations) and PM_10_ (annual mean of daily concentrations; annual mean of the 95th percentile of daily concentrations) within cells of a 4x4 kilometer grid. For each urban unit we calculated a range of concentration parameters for the two pollutants, averaging all cells for each urban unit. As there is a considerable temporal variability in pollutant levels throughout the day and the seasons, in addition to the annual mean we used parameters that illustrate this variety: annual mean of daily 95^th^ percentile and mean of daily winter (NO_2_). The intra-urban level of variability of air pollutant was assessed by calculating a coefficient of variation for each urban unit and each pollutant. These variation coefficients were calculated from estimated air pollution concentrations within cells in a grid of 4 km x 4 km. These heterogeneity indicators contribute to the description of cities’s physical “profile”.

### Statistical processing

Thus a substantial amount of data was mobilized and we analyzed it in an exploratory way. To start the relationships between hospitalization’s rates on the one hand, and all socioeconomic and physical indicators on the other hand, were explored using bivariate statistical analyses (correlations and analysis of variance). Secondly, the purpose was to highlight the complementary roles of different indicators liable to account for health status differences between urban units, and to assess the impact of each of these indicators, *ceteris paribus*. We proceeded in different steps. First we developed multiple regression analyses separately for each family of indicators. We considered four families of socioeconomic indicators: unemployment, education, medical amenity, the size of the urban unit; and three families of physical indicators: climatic and topographic features, pollen and finally ambient air pollution. This first step was developed in order to select the most appropriate indicators in each family and avoid co-linearity problems [94]. Indeed, using measurements relative to different sub-sets of population (for example unemployment for different age groups) on the one hand and to different geographical scales on the other (for example unemployment rate both at the urban scale and at the regional scale), the indicators were collinear. For each family of indicators we selected the ones producing the highest explained variance, eliminating those with co-linearity. The second step consisted in a forward stepwise regression (SAS ©) on all selected indicators. Only classic regressions were developed. Indeed, the number of urban units by region being very small (21 regions for 55 urban units), multi-level analyses were not appropriate. Novelty is in the chosen scale of analysis (urban units) and the combination of explanatory variables derived from different thematic domains and geographical scales, in an exploratory approach.

## Results

**Figure 4:**
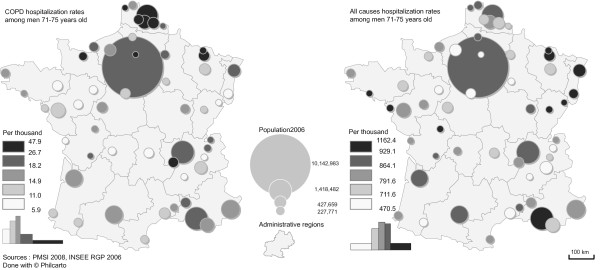
COPD hospitalization rates and overall hospitalization rates among men aged between 71 and 75 in the largest urban units in France (2008).

COPD hospitalization rates among elderly men varied by a factor of eight, from 6 (Chambéry) to 48 per 1,000 individuals (Valenciennes). A spatially structured distribution was observed, with higher hospitalization rates mostly in the urban units in Northern and North-Eastern France, and generally lower rates across the rest of France, especially in the medium-sized urban units (Figure 4). The overall hospitalization rate for all causes ranged from 471 (Annecy) to 1,162^l^ per 1,000 individuals (Saint-Nazaire), with a spatial distribution that was significantly different from that of COPD hospitalizations. Most notably, the Eastern urban units, some smaller Western ones and Marseille indicated high levels, whereas Northern urban units showed a combination of medium and low levels. The largest urban units showed medium to high levels. It appeared clearly that COPD and all hospitalizations show different geographies.

The first step consisted in simple bivariate statistical analyses. They showed that most socioeconomic indicators were highly correlated with COPD hospitalization rates in the urban units (Table 1). As an example, COPD hospitalizations ratio for elderly males were positively and quite strongly correlated (r between 0,3 and 0,5) with the percentage of unemployment, the percentage of persons with no diplomas, and the percentage of non taxable households. On the opposite COPD hospitalizations showed a negative correlation with the percentage of individuals with high diplomas (r=−0,5). Positive associations (r between 0,3 and 0,5) were found with the intra-urban organization indicators such as the coefficient of variation (CV), or the Gini concentration ratio of population with a bachelor or bachelor and two years of university college. Negative correlations (r between −0,3 and −0,5) were found between COPD hospitalization rates and intra-urban organization indicators such as CV or Gini concentration ratio of population with no diploma. No associations were found with the importance of medical amenities or with the size of the urban unit (total number of inhabitants).

**Table 1:** Relationships between hospitalizations and some of the explanatory indicators for COPD and overall hospitalizations

	**Type of indicator**	**COPD hospitalizations**		**Overall hospitalizations**	
		***Signi-ficance level***	***Type of relationship***	***Signi-ficance level***	***Type of relationship***
**Socio-economic indicators**	***Urban unit scale *:**	n.s	no significant relation	n.s	no significant relation
	**Size (number of inhabitants)**				
	***Urban unit scale*** :	0.001	Linear: privileged cities have lower hospitalization rates	n.s	no significant relation
	**Socioeconomic (% unemployment,% of individuals with no diploma, bachelor diploma,% of non taxable households, etc.)**				
			Positive with unemployment, non taxable households (0.3<r<0.5)		
			Negative with bachelor diploma (r=−0.5)		
	***Intra-urban scale***	0.001	Linear positive: cities with concentration of privileged populations have higher hospitalization rates (0.3<r<0.44)	0.001	Linear positive: cities with concentration of privileged populations have higher rates (0.28<r<0.40)
	**Residential intra-urban organization indicators (CV, Gini, Moran of unemployment,% of individuals with no diploma, bachelor diploma,% of non taxable households, etc.)**				
			Linear negative: cities with concentration of deprived populations have lower rates (−0.3<r<−0.48)		
					Linear negative: cities with concentration of deprived populations have lower rates (r=−0.43)
	***Regional scale***	0.001	Linear positive: cities in deprived regions have higher hospitalization rates (0.33<r<0.47)	n.s	no significant relation
	**Socioeconomic (% unemployment, etc.)**				
	***Urban unit scale***	n.s	no significant relation	n.s	no significant relation
	**Access to health amenities**				
**Physical indicators**	***Urban unit scale*** :	0.05	Linear negative with the daily mean temperature in January (r=−0.28)	0.05	no significant relation
	**Climate parameters (temperature, humidity)**				
			Linear positive with the mean annual minimal relative humidity (r=0.34) No significant relation with other climate indicators		
	***Regional scale***	n.s	no significant relation	0.007	Higher hospitalization rates in semi-continental climate ; lower rates in mountain climate
	**Climate zones**				
	***Urban unit scale ***:	0.05	Globally lower hospitalization rates for less polluted locations	n.s	no significant relation
	**Air pollution (ambient levels of PM**_**10**_**, NO**_**2**_**)**				
			Linear positive for PM_10_ (0.28<r<0.30 )		
			Non linear positive for NO_2_		
	***Intra-urban scale ***:	n.s	no significant relation	n.s	no significant relation
	**Air pollution (intra-urban variations of PM**_**10**_**, NO**_**2**_**)**				
	***Urban unit scale***	n.s	no significant relation	n.s	no significant relation
	**Pollen index**				

**Figure 5:**
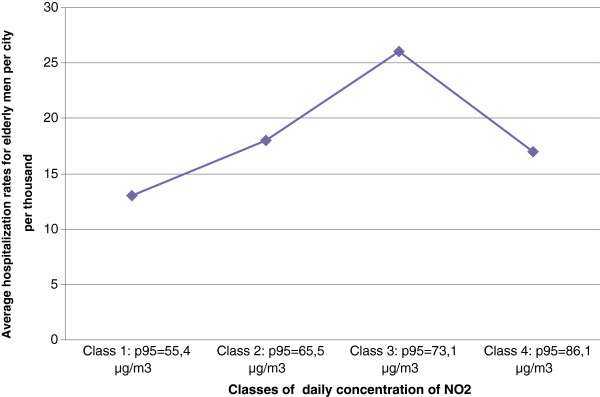
Nonlinear relationship between average COPD hospitalization rates among elderly men and nitrogen dioxide concentration levels (2008).

Associations between COPD hospitalizations ratio for elderly males and physical indicators were much less systematic. Only few were significant, and less than the correlations between COPD hospitalizations and the socioeconomic indicators. There was for example a negative association between COPD hospitalizations with January maximum temperatures (r=−0,28) and positive with humidity (r=0,34). There were no associations with other physical indicators as altitude, pollen or wind. Regarding air pollution, there were a weak positive correlation (r around 0,3) with PM_10_ annual mean of daily concentrations and annual mean of the 95th percentile of daily concentrations. A nonlinear relationships was observed between NO_2_ levels and COPD hospitalization rates. In most urban units, respiratory health status seemed to deteriorate as the NO_2_ level increased. However, the highest NO_2_ concentrations correspond to urban units with moderate COPD hospitalization rates, and not with the highest, as could have been expected (Figure 5).

Conversely, there was almost no relationship between the inter-urban distribution of the benchmark indicator “overall hospitalizations” and the socioeconomic and physical indicators. The only correlations that were statistically significant concerned the CV and Gini coefficient defining the intra-urban distribution of populations according to their levels of education. These correlations were positives, and thus similar to those observed for COPD hospitalizations. Except for these indicators, neither socioeconomic nor physical indicators were correlated with “overall hospitalizations”.

In a second step we developed multiple regressions separately for each family of indicators in order to select the most appropriate for the final analysis. In some cases only one indicator was selected. This was the case for unemployment. The variable producing the highest explained variance was the rate for 15–64 unemployment age group at the regional scale. Once this indicator taken into account, no other indicator belonging to the unemployment family of indicators was significant. In other cases a few indicators were selected. As an example, we found an additive effect of the two pollutants that were investigated, whereby together they accounted for 33% of the inter-urban variations in COPD rates.

In a third step a forward stepwise regression was performed on the selected indicators in preceding step in order to explore the relationship between respiratory health status and the different indicators studied *ceteris paribus*. The analyses revealed a combination of factors that explained the statistics pertaining to health status in the urban units. The model obtained for elderly male combined socioeconomic and physical indicators on different geographical scales: an unemployment indicator on regional scale, a climate indicator on urban scale and two indicators reflecting intra-urban residential organization. This model explained 53% of inter-urban differences in COPD hospitalization rates (Table 2). This R^2^ was high compared to the 27% obtained with the model derived for “overall hospitalizations”, where only two indicators (both relating to the intra-urban socioeconomic organization) were significant. A comparison of the results from these models suggests that socioeconomic and physical factors are both specifically discriminating for respiratory health status. The model residuals showed no spatial autocorrelation. We verified the robustness of these results on the standardized indexes.

**Table 2:** Results for the multiple regression models explaining respectively for “COPD hospitalization” rates and “overall hospitalization” rates

**Explained variable**	**Explanatory variables**	***Coefficient***	***Significance level***
**COPD hospitalization rates for men aged between 71 and 75, calculated as proportion of male population in same age group**	**Intercept**	11	0.24
	**Unemployment rates for 15–64 age group (Regional scale)**	171	0.002
	**Gini coefficient (Intra-urban scale): individuals with bachelor degree and two years of college university study as proportion of overall population**	54	0.023
	**Coefficient of variation (Intra-urban scale) of individuals with no diploma**	−35	0.0006
	**Daily temperature maximum in January (Urban unit scale)**	−0,93	0.037
	**% of explained variance (R**^**2**^**)**	53%	0.0001
**Explained variable**	**Explanatory variables**	***Coefficient***	***Significance level***
**Overall hospitalization rates for men aged between 71 and 75, calculated as proportion of male population in same age group**	**Intercept**	1002	0.0001
	**Gini coefficient (Intra-urban scale): individuals with bachelor degree and two years of college university study as proportion of overall population**	911	0.04
	**Coefficient of variation (census tracts scale) of non-taxable households**	−866	0.001
	**% of explained variance (R**^**2**^**)**	27%	0.002

## Discussion

Lower levels for COPD hospitalization ratios for elderly males tended to be found in cities located in Western and South-Western regions. High levels were mostly found in Northen and North-Eastern regions. This spatial pattern is consistent with that obtained by Delmas and Fuhrman in 2010 [32] on COPD and asthma at the regional scale and by Rican et al. [36] in their 2003 study on respiratory diseases in French urban units with more than 20,000 inhabitants. These authors suggest that there might be an effect of mining and steel manufacturing industry. This is true for several urban units of this study (St-Etienne, Thionville and Béthune). However other urban units like Creil, Caen or Calais that have no such specialization also show high rates. This issue needs to be further investigated.

The geography of both COPD and overall hospitalizations is not specific to any age group, since the standardized indexes show the same patterns. The proxies used to describe urban unit health status could be improved. Indeed, in this study, they were based on hospitalizations alone, which, according to Vigneron [95], only represents half of total health care consumption in France. Tonnellier [96] showed that hospital consultations are more frequent than visits to the family physician in Northern France, as well as in medium-sized and smaller urban units. We intend to conduct further investigations to gain better insight into the inter-urban differences in respiratory health status, by considering both hospitalizations and visits to family physicians and specialists.

Multiple regression analyses were used to explain inter-urban differences for both COPD and overall hospitalization rates. Many statistically significant relationships were observed between COPD rates and the candidate explanatory socioeconomic and physical dimensions. The COPD models combined indicators associated with both physical and socioeconomic characteristics and involved different geographical scales. Conversely, very few strong relationships were observed for overall hospitalization rates. This reinforces our hypothesis that respiratory health status exhibits specific inter-urban patterns.

Our first hypothesis that the socioeconomic and physical dimensions both need to be considered in order to provide a statistical explanation for COPD differences across urban units is not clearly borne out. Our results showed that inter-urban differences in respiratory health status are more systematically related to socioeconomic factors than physical ones. The relationships between health and socioeconomic indicators were similar to those established using a deprivation index in intra-urban studies [36,53,97,98]. These results are also consistent with studies on socioeconomic inequalities and health carried out at different geographical scales, for example by Leclerc et al. [53] at a regional scale, Rican, et al. [35,36] at the city and regional scale, Blomgren et al. [99] at the regional scale, and Macintyre et al. [98] at the small-area (intra-urban) scale.

The positive correlation between average annual humidity and COPD hospitalization rates confirmed the evidence reported in the literature [11,76,80,81]. One of the climate parameters integrated into the model contributed clearly to explaining inter-urban differences: the annual mean of maximum temperature in January was negatively associated with hospital admission rates for COPD. This is consistent with results that have shown the existence of a relationship between temperatures and asthma [11,75-77,79]. However, for asthma prevalence the direction of the relationship is positive with annual temperature means [11,77,79] or lowest monthly temperature means in the ISAAC study [76]. Our results are different, as they showed that the higher the temperature in January, the lower the COPD rates. A direct comparison of results is not possible, because in the literature asthma was the object of study and not COPD prevalence, and the age groups observed were different: children [76], young adults or adults [11,75,77,79]. However, the negative relationship between temperature and hospital admissions for COPD clearly appears in temporal studies and it is in January that the maxima of monthly hospital admissions are observed [78]. There is no confirmation in the literature that the association between temperature and respiratory health outcomes reflects a causal relationship, or that correlations are a result of indirect relationships and linked to other factors, like air pollution levels.

The adverse effect of air pollution on health has been well established in literature, and our results are consistent with only some of this evidence. COPD hospitalization rates were significantly, but weakly correlated, with all parameters describing PM_10_ concentrations in the urban units. This is consistent with the literature, where COPD appears clearly linked with PM_10_ ambient levels [7,39,88-92]. The non linearity of the relation between NO_2_ levels and COPD hospitalization rates of elderly males could be attributed to a compensatory effect brought about by the higher socioeconomic status of these larger urban units, which are also characterized by the highest air pollution levels. This is consistent with the conclusions of Cakmak et al. [100] in their study on the way community income and education modify the effect of gaseous air pollution on respiratory hospitalizations. Indeed, based on analyses of daily time series for respiratory hospitalizations and daily concentrations of NO_2_ concentrations in 10 large Canadian cities, they found that the effect of both NO_2_ and a combination of pollutants was stronger with decreasing levels of household income.

However, in our regression model the air pollutant indicators did not appear. In our study, socioeconomic indicators seem to obliterate the effect of nitrogen dioxide variations on COPD hospitalizations. This partly invalidates our earlier hypothesis regarding the contribution of atmospheric pollution to the inter-urban differences in COPD rates: once socioeconomic and climate indicators are taken into account, the concentration of pollutants such as nitrogen dioxide and particulate matter no longer provides a statistical explanation for variations in COPD rates across urban units.

Our second hypothesis, that explanatory factors should be considered on different spatial scales to account for differences in respiratory health status among urban units, was confirmed. The most innovative results were those that involved the use of regional and intra-urban indicators. First, when combined with other variables, unemployment showed a more significant contribution on regional than on urban scale. Thus, an urban unit’s respiratory health status could be partially explained by its belonging to a regional economic context. This result is consistent with the findings of Rican et al. [36,53,97,98], who reported that the regional component of respiratory mortality variations in France is highly significant. It is also consistent with Jusot’s result [101] that the individual mortality risk among male adults in France includes a regional component. Secondly, an intra-urban organization effect was clearly observed. In the literature, it has been shown that there are positive associations between marked racial residential segregation and mortality rates or tobacco risk on city scale [23-25]. Several explanations have been put forward: segregation may limit the social and economic opportunities for deprived groups, worsen their deprivation, and in turn lower the city’s overall health status; similarly, residential segregation could restrict mobility and access to health services, and consequently affect a city’s health status. We expected to observe the same overall effect of marked residential differentiation on health status. However, the statistical results proved far from simple: residential organization of the undereducated population within the urban unit was found to correlate negatively with COPD rates on urban unit scale; conversely, the residential organization of highly educated populations was found to correlate positively with COPD rates. This seems to imply that the spatial concentration of less educated populations is associated with better respiratory health status, whereas spatial concentration of more highly educated populations is associated with lower respiratory health status at the urban unit scale. Even though the number of IRIS census tracts varied between urban units, no statistical effect arising from differences in urban unit sizes seems to have affected these results. The relationships with residential organization are then partially coherent with the conclusions of various studies cited above, in the sense that the results are far from simple and will require further investigation.

## Conclusion

It is important to gain a better understanding of the spatial patterns of urban respiratory health problems, since respiratory health is a growing cause of morbidity worldwide. It is reported as being dependent on both socioeconomic and physical environmental factors, more specifically pollutants. Our aim is to explore these hypotheses at the inter-urban scale. We do not aim to provide any predictive model about a disease outcome and environmental exposure, neither to establish any causality, at the level of the city or at the individual level.

First the study shows surprising results for the contribution of pollution factors. Indeed, differences in respiratory health among cities related largely to socioeconomic factors and only marginally to pollution. Second, the study underlines that broader contextual effects come into play. Indeed it appeared that differences in health respiratory status between cities arose from the economic regional context (in terms of unemployment ): thus characteristics observed at the regional scale contribute to explain inter-urban differences in respiratory health status. Third, inter-urban inequalities in health status are related to intra-urban organizational factors, such as differentiated residential locations of specific population groups (highly qualified groups or non-qualified groups). In order for public policy makers to plan “healthy cities” and thus improve overall public health, it is essential to gain a better understanding of the complex and probably very interdependent factors that contribute to the development of spatial health variations. Indeed, the hypotheses examined - 1) that two cities with similar socioeconomic and physical environment characteristics but different degrees of intra-urban residential organization could differ in terms of respiratory health status and 2) that two cities with the same socioeconomic and physical characteristics but situated in different regional contexts may not have the same respiratory health profiles - are confirmed.

## Endnotes

^a^ Our aim is by no means to state that an inhabitant in city X has a greater or lesser risk of contracting a respiratory disease than an inhabitant in city Y.

^b^ Each shade of grey represents the density of presence of a certain type of population (highly educated persons for example): dark-grey stands for a high density, pale-grey for a low density. The examples depict common intra-urban patterns of residential organization. These differences form segregation patterns within the city: from center towards periphery in the first graph, marked contrast in the second (in these two graphs intra-urban differentiation is high), lesser contrast in the third and fourth graph (intra-urban differentiation is lower).

^c^ An Urban Unit is a municipality or group of municipalities with at least 2,000 inhabitants. An urban unit is a continuous built up area in the sense that there is less than 200 meters between two buildings (Insee web site, 2013).

^d^ The Functional Urban Area can be defined as travel-to-work area. Principally it is an agglomeration of work places attracting the work force from the surrounding area.

^e^ PMSI: Programme Médical Système d’Information(Source: Agence Technique de l’Information Hospitalière [http://www.atih.sante.fr]).

^f^ INSEE (“Institut National de la Statistique et des Etudes Economiques”) is the French National Institute of Statistics and Economic Studies (http://www.insee.fr).

^g^ Data is derived from the “Base Permanente des Equipements” at INSEE and originally from ADELI (Automatisation DES LIstes), which keeps records of all registered health practitioners in France.

^h^ These census tracts are statistical units defined by INSEE, known as IRIS (Ilots Regroupés pour des Indicateurs Statistiques). They roughly comprise between 1,800 and 5,000 individuals.

^i^ “Réseau National de Surveillance Aérobiologique”.

^j^ The ten pollen species used to build the aerobiological indicator are : 

Trees: alder (*Alnus*), cypress (*Cupressus*), oak (*Quercus*), ash (*Fraxinus*),birch (*Betula*), plane (*Platanus*), olive (*Olea*)

Grasses (*Family Poaceae*): no distinction was made between species of grass

Weeds: ragweed (*Ambrosia*), nettle/parietaria (*Urticaceae*)

^k^ ADEME, “Agence de l’Environnement et de la Maîtrise de l’Energie”, is the French Environment and Energy Management Agency[http://www2.ademe.fr].

^l^ There can be more than 1,000 hospitalization for 1,000 individuals as one individual can have paid more than one visit to the hospital in a year.

## Abbreviations

COPD: Chronic Obstructive Pulmonary Disease; CV: Coefficient of variation; IRIS: Ilots Regroupés pour des Indicateurs Statistiques: These census tracts are statistical units defined by INSEE, known as IRIS. They roughly comprise between 1,800 and 5,000 inhabitants; NO2: Nitrogen Dioxyde; O3: Ozone; PMSI: Programme Médical des Systèmes d’Information; PM10: Particulate Matter, diameter inferior to 10 μm; RNSA: Réseau National de Surveillance Aérobiologique.

## Competing interest

The authors have no conflict of interest to declare.

## Authors’ contributions

CAL, SBM, HM, LS conceived the study, participated in its design and coordination and helped to draft the manuscript. All authors read and approved the final manuscript.

## Supplementary Material

Additional file 1 Indicators and measurements. Click here for file
