# Supplementary material for: Gaining a better understanding of respiratory health inequalities among cities: An ecological case study on elderly males in the larger French cities
Source: Int J Health Geogr. 2013 Apr 10;12:19. doi: 10.1186/1476-072X-12-19 (PMC3735046; doi:10.1186/1476-072X-12-19)
Supplement: Additional file 1 — Indicators and measurements. [file 1476-072X-12-19-S1.docx]

|  | **Scale** | **Indicator** | **mean** | **min** | **max** | **std** |
| --- | --- | --- | --- | --- | --- | --- |
| **Socioeconomic indicators** | Urban unit | Standardized ratio, for COPD hospitalization, for males over 65 years old | 0.95 | 0.38 | 2.76 | 0.46 |
|  |  | COPD hospitalization rate for males aged 71 to 75 years (for 1000) | 18.66 | 5.93 | 47.88 | 9.65 |
|  |  | Standardized ratio, overall hospitalization, for males over 65 years old | 0.77 | 0.48 | 1.14 | 0.13 |
|  |  | Overall hospitalization rate for males aged 71 to 75 years (for 1000) | 828.1 | 470.5 | 1,162.4 | 149.2 |
|  |  | Number of resident (thousand) | 507 | 96.5 | 10,143 | 1359 |
|  |  | Unemployment rate (15-64 years) | 0.13 | 0.08 | 0.22 | 0.03 |
|  |  | Unemployment rate (15-24 years) | 0.23 | 0.14 | 0.43 | 0.06 |
|  |  | Unemployment rate (25-54 years) | 0.12 | 0.08 | 0.19 | 0.02 |
|  |  | Unemployment rate (55-64 years) | 0.09 | 0.05 | 0.14 | 0.02 |
|  | Intra-urban scale | Coefficient of variation of unemployment rates across IRIS census tracts in the urban unit (15-64 years) | 0.53 | 0.34 | 0.68 | 0.08 |
|  |  | Gini index on intra-urban unemployment across IRIS census tracts (15-64 years)(over total working pop.) | 0.25 | 0.18 | 0.31 | 0.03 |
|  |  | Moran autocorrelation index - on intra-urban unemployment rates across IRIS census tracts (15-64 years)(over total working pop.) | 0.28 | -0.05 | 0.54 | 0.13 |
|  |  | Coefficient of variation of unemployment rates across census tracts in the urban unit (15-64 years) | 0.47 | 0.25 | 0.78 | 0.10 |
|  |  | Gini index - unemployment across census tracts in the urban unit (15-24 years) (over total working pop) | 0.25 | 0.12 | 0.33 | 0.04 |
|  |  | Moran autocorrelation index - unemployment rate in the urban unit (15-24 years) (over total working pop) | 0.18 | -0.14 | 0.51 | 0.12 |
|  |  | Coefficient of variation of unemployment rates across the urban unit census tracts (25-54 years) | 0.57 | 0.36 | 0.71 | 0.08 |
|  |  | Gini index - unemployment across census tracts in the urban unit(25-54 years) (over total <orking piop) | 0.27 | 0.19 | 0.33 | 0.03 |
|  |  | Moran autocorrelation index - on intra-urban unemployment rates across IRIS census tracts (25-54 years)(over total working pop.) | 0.28 | -0.02 | 0.56 | 0.13 |
|  |  | Coefficient of variation of unemployment rates across census tracts in the urban unit (55-64 years) | 0.70 | 0.43 | 1.16 | 0.13 |
|  |  | Gini index - unemployment across census tracts in the urban unit (55-64 years)(over total working pop) | 0.36 | 0.25 | 0.48 | 0.05 |
|  |  | Moran autocorrelation index - on intra-urban unemployment rates across IRIS census tracts (55-64 years)(over total working population) | 0.11 | -0.14 | 0.39 | 0.11 |
|  | Regional scale | Unemployment rate (15-64 years) | 0.11 | 0.09 | 0.15 | 0.02 |
|  |  | Unemployment rate (15-24 years) | 0.22 | 0.17 | 0.32 | 0.04 |
|  |  | Unemployment rate (25-54 years) | 0.12 | 0.09 | 0.16 | 0.02 |
|  |  | Unemployment rate (55-64 years) | 0.09 | 0.06 | 0.12 | 0.02 |

|  | **Scale** | **Indicator** | **mean** | **min** | **max** | **std** |
| --- | --- | --- | --- | --- | --- | --- |

| **Socioeconomic indicators** | Urban unit | Rate of population 15 and over not attending schools and with no diploma | 0.19 | 0.11 | 0.30 | 0.04 |
| --- | --- | --- | --- | --- | --- | --- |
|  |  | Rate of population 15 and over not attending schools with a bachelor degree or equivalent | 0.15 | 0.12 | 0.17 | 0.01 |
|  |  | Rate of population 15 and over not attending schools with a bachelor degree and two years of studies, university college | 0.24 | 0.13 | 0.36 | 0.06 |
|  | Intra-urban scale | Coefficient of variation across census tracts in the urban unit for the proportion of persons no longer attending school, with no diploma | 0.53 | 0.28 | 0.79 | 0.11 |
|  |  | Gini index - persons over 15 no longer attending school, with no diploma | 0.25 | 0.16 | 0.34 | 0.04 |
|  |  | Moran autocorrelation index - rates of population over 15 no longer in school, no diploma | 0.30 | 0.00 | 0.60 | 0.14 |
|  |  | Coefficient of variation across census tracts in the urban unit for rates of population over 15 not attending school, with bachelor degree or equivalent diploma | 0.24 | 0.11 | 0.43 | 0.06 |
|  |  | Gini index across census tracts in the urban unit for population over 15 not attending school, with bachelor degree or equivalent diploma | 0.11 | 0.06 | 0.16 | 0.02 |
|  |  | Moran autocorrelation index across census tracts in the urban unit for rates of population over 15 not attending school, with bachelor degree or equivalent diploma | 0.17 | -0.07 | 0.42 | 0.11 |
|  |  | Coefficient of variation across census tracts in the urban unit, for rates of population not attending school, with bachelor degree plus two years of university college (over total pop 15+ not in school) | 0.37 | 0.21 | 0.67 | 0.10 |
|  |  | Gini index across census tracts in the urban unit, for population not attending school, with bachelor degree plus two years of university college | 0.18 | 0.11 | 0.29 | 0.04 |
|  |  | Moran autocorrelation index across census tracts in the urban unit, for rates of population not attending school, with bachelor degree plus two years of university college | 0.29 | 0.02 | 0.59 | 0.13 |
|  | Regional scale | Rates of population over 15 not attending school with no diploma | 0.20 | 0.15 | 0.25 | 0.02 |
|  |  | Rates of population over 15 not attending school, with a Bachelor degree or equivalent | 0.15 | 0.13 | 0.16 | 0.01 |
|  |  | Rates of population over 15 not attending school, with a Bachelor degree and at least two more years of university college | 0.20 | 0.16 | 0.33 | 0.03 |

|  | **Scale** | **Indicator** | **mean** | **min** | **max** | **std** |
| --- | --- | --- | --- | --- | --- | --- |

| **Socioeconomic indicators** | Urban unit | Proportion of households per urban unit not liable for income tax | 40.81 | 27.36 | 54.29 | 5.57 |
| --- | --- | --- | --- | --- | --- | --- |
|  | Intra-urban scale | Coefficient of variation across the census tracts in the urban unit for the proportion of households not liable for income tax | 0.31 | 0.14 | 0.59 | 0.07 |
|  |  | Gini index across the census tracts in the urban unit for the proportion of households not liable for income tax | 0.19 | 0.10 | 0.32 | 0.04 |
|  |  | Moran autocorrelation index across the census tracts in the urban unit for the proportion of households not liable for income tax | 0.22 | -0.06 | 0.46 | 0.12 |
|  |  |  |  |  |  |  |
|  | Urban unit | Number of general practitioners per 10,000 inhabitant | 12.64 | 9.11 | 16.90 | 2.09 |
|  |  | Number of pneumonologists per 10,000 inhabitant | 0.35 | 0.08 | 1.21 | 0.18 |
| Physical indicators | Urban unit | Daily temperature minima in January (C°) | 0.93 | -3.00 | 6.00 | 1.93 |
|  |  | Daily temperature maxima in January (C°) | 6.96 | 3.00 | 13.00 | 2.47 |
|  |  | Daily temperature minima in July (C°) | 13.62 | 11.00 | 19.00 | 2.02 |
|  |  | Daily temperature maxima in July (C°) | 24.15 | 19.00 | 29.00 | 2.35 |
|  |  | Average number of hot days per year (> 25°C) | 8.75 | 1.00 | 31.00 | 6.93 |
|  |  | Annual mean of outdoor relative humidity minima | 58.60 | 45.00 | 67.00 | 5.48 |
|  |  | Average number of foggy days per year | 47.42 | 1.00 | 101.0 | 23.91 |
|  |  | Average number of days with strong wind per year (wind > 57 km/h) | 49.16 | 20.00 | 132.0 | 27.35 |

|  | **Scale** | **Indicator** | **mean** | **min** | **max** | **std** |
| --- | --- | --- | --- | --- | --- | --- |

| Physical indicators | Regional scale | Climatic zone : oceanic, oceanic altered, continental, mediterranean, mountain |  |  |  |  |
| --- | --- | --- | --- | --- | --- | --- |
|  | Urban unit | Altitude of the urban unit center (meters) | 157.3 | 3.82 | 569.9 | 150.4 |
|  | Intra-urban scale | Relative variation in altitude in the urban unit (2(X_max_ – X_min_)/(X_max_ + X_min_) | 140.1 | 4.00 | 582.0 | 148.3 |
|  | Urban unit | Aerobiological index of the urban unit | 15.56 | 12.00 | 23.00 | 3.25 |
|  |  | Annual mean of daily concentrations of NO_2_ (µg/m^3^) | 20.13 | 11.73 | 28.13 | 4.18 |
|  |  | Annual mean of the 95th percentile of daily concentrations NO_2_ (µg/m^3^) | 126.4 | 98.50 | 164.2 | 17.58 |
|  |  | Winter (Oct-April) mean daily average concentrations of NO_2_ (µg/m^3^) | 26.09 | 15.47 | 38.00 | 5.70 |
|  |  | Annual mean of the daily average concentrations of PM_10_ (µg/m^3^) | 22.40 | 17.10 | 30.87 | 3.49 |
|  |  | Annual mean of the 95th percentile of daily concentrations PM_10_ (µg/m^3^) | 84.50 | 57.54 | 121.5 | 16.34 |
|  | Intra-urban scale | Coefficient of intra-urban spatial varition of annual mean NO_2_ (µg/m^3^) | 0.11 | 0.06 | 0.24 | 0.04 |
|  |  | Coefficient of intraurban spatial varition of annual mean PM_10_ (µg/m^3^) | 0.02 | 0.00 | 0.04 | 0.01 |
